# Comparison of standardized diaphragm ultrasound measurement and conventional methods for predicting weaning failure: a prospective observational study

**DOI:** 10.1007/s00540-026-03655-6

**Published:** 2026-01-24

**Authors:** Yener Aksoy, Asu Ozgultekin, Yelda Balık, Osman Ekinci

**Affiliations:** 1 Department of Anesthesiology and Intensive Care, Sancaktepe Sehit Prof.Dr. Ilhan Varank Training And Research Hospital, University of Health Sciences, Emek Neighborhood, Namik Kemal Street, Number:54, 34785 Istanbul, Turkey; 2 Department of Anesthesiology and Intensive Care, Haydarpasa Numune Training and Research Hospital, University of Health Sciences, 34674 Istanbul, Turkey

**Keywords:** Diaphragm, Ultrasound, Mechanical ventilation, Weaning, Extubation

## Abstract

**Purpose:**

Weaning from invasive mechanical ventilation (IMV) is challenging and has multiple causes. The diaphragm is the main respiratory muscle for inspiration. This prospective study aimed to determine the value of standardized diaphragm ultrasound (DUS) measurements [diaphragm excursion (DE), diaphragm thickness fraction (DTF), diaphragmatic rapid shallow breathing index (D-RSBI), rapid shallow diaphragmatic index (RSDI)] in predicting extubation success in intensive care patients, both individually and in combination with conventional indices [rapid shallow breathing index (RSBI), dynamic compliance (Cdyn), airway occlusion pressure, semi-quantitative cough strength score]. To isolate diaphragm contribution, only neurologically intact patients (Glasgow Coma Scale > 14) with adequate airway protection reflexes were included. The second aim was to examine the relationship between IMV and DUS measurements.

**Methods:**

151 patients on IMV for > 24 h and eligible for spontaneous breathing trial (SBT) were evaluated. Following exclusion criteria, patients underwent SBT in pressure support ventilation mode (positive end-expiratory pressure 5 cmH_2_O, pressure support 8 cmH_2_O). During SBT, mechanical ventilation parameters and diaphragm ultrasound measurements were recorded. Extubation failure was defined as need for reintubation or non-invasive ventilation.

**Results:**

DE and DTF were significantly higher, D-RSBI was lower in patients with successful extubation. There was no difference in RSDI. Multivariate logistic regression was statistically significant, odds ratios (10.018, 1.109, 1.094) were found for DE, DTF, Cdyn, respectively. The only significant correlation between IMV and DUS was DTF-tidal volume (*r* = − 0.500).

**Conclusion:**

A standardized multiparametric model, combining DUS with conventional indices, provides moderate predictive accuracy for extubation success. Integrating DUS into weaning protocols can improve extubation readiness.

**Supplementary Information:**

The online version contains supplementary material available at https://doi.org/10.1007/s00540-026-03655-6.

## Introductıon

Weaning from invasive mechanical ventilation is a critical process that must be timed appropriately to optimize clinical outcomes. Early weaning attempts, result in extubation failure and reintubation, while delaying the process puts patients at the risk of serious complications such as ventilator-associated pneumonia, barotrauma, and ventilator-induced diaphragm dysfunction (VIDD). Both early and late weaning are associated with increased mortality, morbidity, and healthcare costs [1, 2].

The rapid shallow breathing index (RSBI) is the most common conventional parameter used in clinical practice to predict extubation success. However, RSBI alone has limited sensitivity and specificity in determining extubation success. Current evidence does not support its standalone use, and further studies are needed to assess its efficacy as a part of multiparametric approach [3].

Diaphragm muscle is the primary inspiratory muscle that develops atrophy within the first 24 h in patients on IMV. Diaphragm ultrasound has emerged as a non-invasive, bedside tool to directly evaluate diaphragmatic function. DE and DTF are ultrasound measurements that reflect muscle’s displacement and contractility [4].

However, lack of standardization in measurement techniques is an important obstacle to synthesize the results in the literature. Ventilation mode used during spontaneous breathing trial (SBT) [synchronized intermittent mandatory ventilation (SIMV), pressure supported ventilation (PSV), T-tube] [5–7], positive end-expiratory pressure (PEEP) levels during measurement [8–10], patient positioning [11], side of the measured diaphragm (right or left) [12], border for diaphragm thickness measurements using either middle part of the pleura and peritoneal sheets [13] or boundaries of the diaphragm muscle and pleura/peritoneum [14] complicates comparison across studies. Moreover, since the effects of these variables on the measurement results are still unclear, these critical details are not always specified in the method sections of the articles, which worsens the existing heterogeneity.

In addition, patients with a Glasgow Coma Scale (GCS) of 13–14 are at significantly higher risk of intubation compared to patients with a GCS of 15, because even borderline neurological dysfunction may impair airway protection or effective secretion clearance [15]. Previous studies have demonstrated that PEEP levels significantly alter anatomical and physiological functioning of diaphragm. In healthy volunteers undergoing non-invasive ventilation, increasing PEEP from 2 to 15 cmH_2_O resulted in increased diaphragm thickness, caudal displacement, shortening of muscle fibers in the apposition zone, and, more importantly, a 48% decrease in neuromechanical efficiency [8]. Similarly, increasing PEEP from 0 to 10 cmH_2_O has been associated with increases in end-inspiratory diaphragm thickness (TDI), end-expiratory diaphragm thickness (TDE), diaphragm thickening fraction (DTF), diaphragm excursion (DE), and tidal volume [9]. Although the correlation between DE and tidal volume is significant in healthy individuals, this relationship becomes substantially attenuated in intubated patients due to the confounding effects of PEEP [10]. To minimize this confounding effects and isolate diaphragmatic function, we included only patients with a GCS of 15 (GCS > 14) and intact airway reflexes in the study and applied a fixed PEEP and pressure support protocol. To our knowledge, this is the first study to evaluate diaphragm ultrasound parameters using a standardized protocol with fixed PEEP and pressure support in critically ill with GCS of 15 who were planned for extubation in intensive care unit.

Furthermore, successful extubation is multifactorial, depending not only on inspiratory muscle strength, but also on overall respiratory mechanics, central drive, and airway protective reflexes. For this reason, we aimed to assess the predictive value of standardized diaphragm ultrasound measurements (DE, DTF, D-RSBI, RSDI) for extubation outcomes both individually and combined with conventional weaning parameters [RSBI, dynamic compliance (Cdyn), airway occlusion pressure (P0.1), semi-quantitative cough strength score (SCSS)] for extubation outcomes in intensive care unit (ICU) patients. We also investigated correlations between mechanical ventilation variables and diaphragm ultrasound metrics, hypothesizing that a multiparametric standardized model integrating these parameters would predict extubation success more accurately than any single measure alone.

## Methods

### Study design and ethics

This prospective observational study was conducted in a general intensive care unit of the institution in accordance with the protocol approved by the Haydarpaşa numune training and research hospital clinical research ethics committee, university of health sciences (Approval No: HNEAH-KAEK 2023/82, Approval date: May 8, 2023). Patient enrollment took place between June 1, 2023, and December 1, 2023. Patients with GCS > 14 (GCS = 15) who had decision-making capacity (awake, able to obey commands) were included in the study. Patients and their next of kin were given comprehensive verbal information about the purpose of the study, the procedures, the potential risks, and the benefits, and who wished to read the informed consent form themselves were given sufficient time. After it was clearly explained that participation was voluntary and withdrawal was possible at any time without affecting their treatment, written informed consent was obtained from all participants in accordance with the declaration of Helsinki.

### Participants

We enrolled postoperative or general intensive care adult patients (≥ 18 years) who had been on IMV for ≥ 24 h and were deemed ready for extubation by the attending intensivist.

- **Inclusion criteria for extubation readiness:**
  o Reversal of the reason of intubation.
  o Glasgow Coma Scale > 14 with adequate consciousness and cough reflex.
  o Ramsay Sedation Scale 1–3.
  o Fraction of inspired oxygen (FiO_2_) < 0.4 and PEEP < 8 cmH_2_O.
  o PaO_2_ (partial pressure of arterial oxygen)/FiO_2_ > 150 mmHg.
  o Mean arterial pressure > 60 mmHg (without escalating vasopressors).
  o Urine output > 0.5 mL/kg/h.

Patients were excluded if their diaphragm could not be visualized via ultrasound, if body mass index (BMI) > 35 kg/m^2^, if pregnant, with known diaphragmatic or phrenic nerve injury, neuromuscular disease, pneumothorax, pneumomediastinum, empyema, chest trauma, or recent chest tube placement.

Patients meeting criteria underwent a spontaneous breathing trial (SBT) in pressure support mode with PEEP 5 of cmH_2_O and pressure support of 8 cmH_2_O. The SBT was continued for up to 2 h unless failure criteria occurred. The trial was terminated early and deemed a SBT failure if any of the following occurred. Baseline values refer to measurements taken immediately before SBT. The failure criteria were categorized as follows:

- **Gas exchange failure:**
  o Peripheral oxygen saturation (SpO_2_) < 90% with fraction of inspired oxygen (FiO_2_) of ≤ 0.4.
  o Partial pressure of arterial oxygen (PaO_2_) < 60 mmHg.
  o Partial pressure of arterial carbon dioxide (PaCO_2_) > 50 mmHg, or an increase of > 8 mmHg from the pre-SBT baseline.
  o Arterial pH < 7.20, or a decrease of > 0.07 from the pre-SBT baseline.
- **Hemodynamic instability:**
  o Heart rate > 140 beats/min, or a sustained increase of > 20% from baseline.
  o Systolic blood pressure > 180 mmHg or < 90 mmHg.
- **Increased work of breathing or distress:**
  o Respiratory rate > 30 breaths/min, or an increase of > 50% from baseline.
  o Objective signs of respiratory distress, including the use of accessory muscles, paradoxical abdominal movements.
  o Altered mental status (e.g., agitation: RASS ≥ + 2; somnolence: RASS ≤ − 3; or severe anxiety)

### Diaphragm ultrasound and RSBI

Measurements were obtained in a semi-recumbent position (20–40° head elevation) during the first hour of the SBT. The right hemidiaphragm was assessed by a single trained intensivist using a convex probe (2–4 MHz) that was placed in the right subcostal area along the mid-clavicular line (aiming at the liver-diaphragm interface) to measure excursion (DE), and a linear probe (7–10 MHz) that was placed at the anterior to mid-axillary line in the 8–10th intercostal spaces for thickness measurement. The diaphragm muscle appears as a hypoechoic structure between the hyperechoic peritoneum and pleura on ultrasound. DTF was calculated as [(thickness at end-inspiration—thickness at end-expiration)/thickness at end-expiration] × 100. Thickness measurements at end-inspiration and end-expiration were taken at the midpoint between pleural and peritoneal membranes. The average of three consecutive respiratory cycles was recorded for both excursion and thickness measures. Respiratory rate (RR) and tidal volume (TV) during SBT were recorded from the ventilator. From these, we computed D-RSBI = RR/DE and RSDI = RSBI/DE (where RSBI = RR/TV).

Each parameter’s ability to predict extubation success was evaluated individually.

- 1-These parameters are:
  - Diaphragm excursion (DE).
  - Diaphragm thickness fraction (DTF) = [(thickness at end-inspiration–thickness at end-expiration)/thickness at end-expiration] × 100.
- 2-Derived indices included:
  - Diaphragmatic rapid shallow breathing index (D-RSBI) = RR/DE.
  - Rapid shallow diaphragmatic index (RSDI) = RSBI/DE.

### Conventional parameters

Conventional parameters were recorded including RSBI, respiratory rate (RR), tidal volume (VT), minute ventilation (MV), airway occlusion pressure (P0.1), dynamic compliance (Cdyn), semi-quantitative cough strength score (SCSS, scale 0–5), sequential organ failure assessment (SOFA) score on the day of extubation, relevant laboratory values (phosphorus, total and ionized calcium, magnesium), duration of intubation, age, and gender.

The semi-quantitative cough strength score (SCSS) was evaluated by a single observer and defined as follows: 0 = no cough on command; 1 = air from the endotracheal tube on command but no audible cough; 2 = barely audible cough; 3 = clearly audible cough; 4 = stronger cough; 5 = repeated strong coughs.

### Definition of extubation failure

**Fig. 1:**
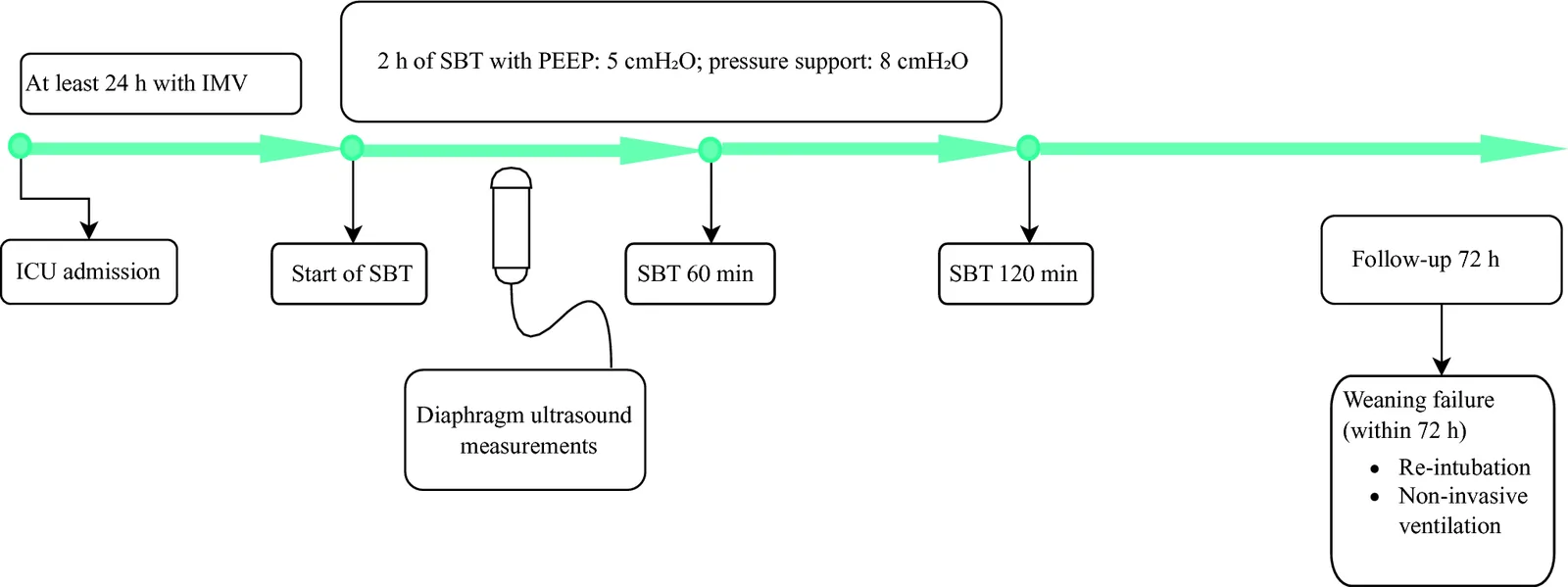
Timeline of the study. *IMV* invasive mechanical ventilation, *PEEP* positive end-expiratory pressure, *NIV* non-invasive ventilation, *SBT* spontaneous breathing trial, *ICU* intensive care unit

Extubation failure was defined as the need for reintubation or non-invasive ventilation (NIV) within 72 h after a planned extubation (Fig. 1).

We determined the sensitivity, specificity, and optimal cut-off values for each diaphragm ultrasound measurement that was statistically significant in predicting extubation success. We then constructed a multivariate logistic regression model for extubation success using the parameters found to be significant, and analyzed each parameter’s contribution to the model. In addition, correlations between diaphragm ultrasound findings and mechanical ventilation parameters were assessed.

### Assessment of ultrasonographic indices’ reproducibility

To assess the intra-observer reliability of diaphragm ultrasound measurements, the video clips (cine-loops) of 34 randomly selected patients were saved. The same observer re-analyzed these clips after a 2-week interval to minimize recall bias. During the second assessment, the clips were reviewed in a random order, and the observer was blinded to the initial measurements and patient identities.

Intra-observer reliability was assessed using a two-way mixed-effects, absolute agreement, single measures model to calculate the ıntraclass correlation coefficient (ICC) and its corresponding 95% confidence interval (CI). Absolute reliability metrics were determined, including the pooled standard deviation (PooledSD), the standard error of measurement (SEM), and the minimal detectable change (MDC) at the 95% confidence level. ICC values were interpreted as poor (< 0.50), moderate (0.50–0.75), good (0.75–0.90), or excellent (> 0.90). Statistical significance was evaluated at the *p* < 0.05 level.

### Statistical analysis

IBM SPSS Statistics 22 was used for all analyses. The conformity of the parameters to normal distribution was evaluated via Kolmogorov–Smirnov and Shapiro–Wilks tests. In addition to descriptive statistics (minimum, maximum, mean, standard deviation, median, frequency), we used Student’s *t* test to compare normally distributed continuous variables between 2 groups, and the Mann–Whitney *U* test for non-normally distributed variables. Continuity (Yates) correction was used for comparison of qualitative data. Cohen’s d was used to determine the effect size. Optimal cut-off points were determined using receiver operating characteristic (ROC) curve analysis. Pearson’s correlation analysis was used to examine the relationships between parameters that conformed to a normal distribution, and Spearman’s rho correlation analysis was used to examine the relationships between parameters that did not conform to a normal distribution. We assessed multicollinearity among significant predictor variables using Variance Inflation Factors (VIF) and the condition index. Highly correlated ROC curves were compared using the DeLong test. A multivariate logistic regression was performed for extubation outcome prediction. Statistical significance was evaluated at the *p* < 0.05 level.

## Results

**Fig. 2:**
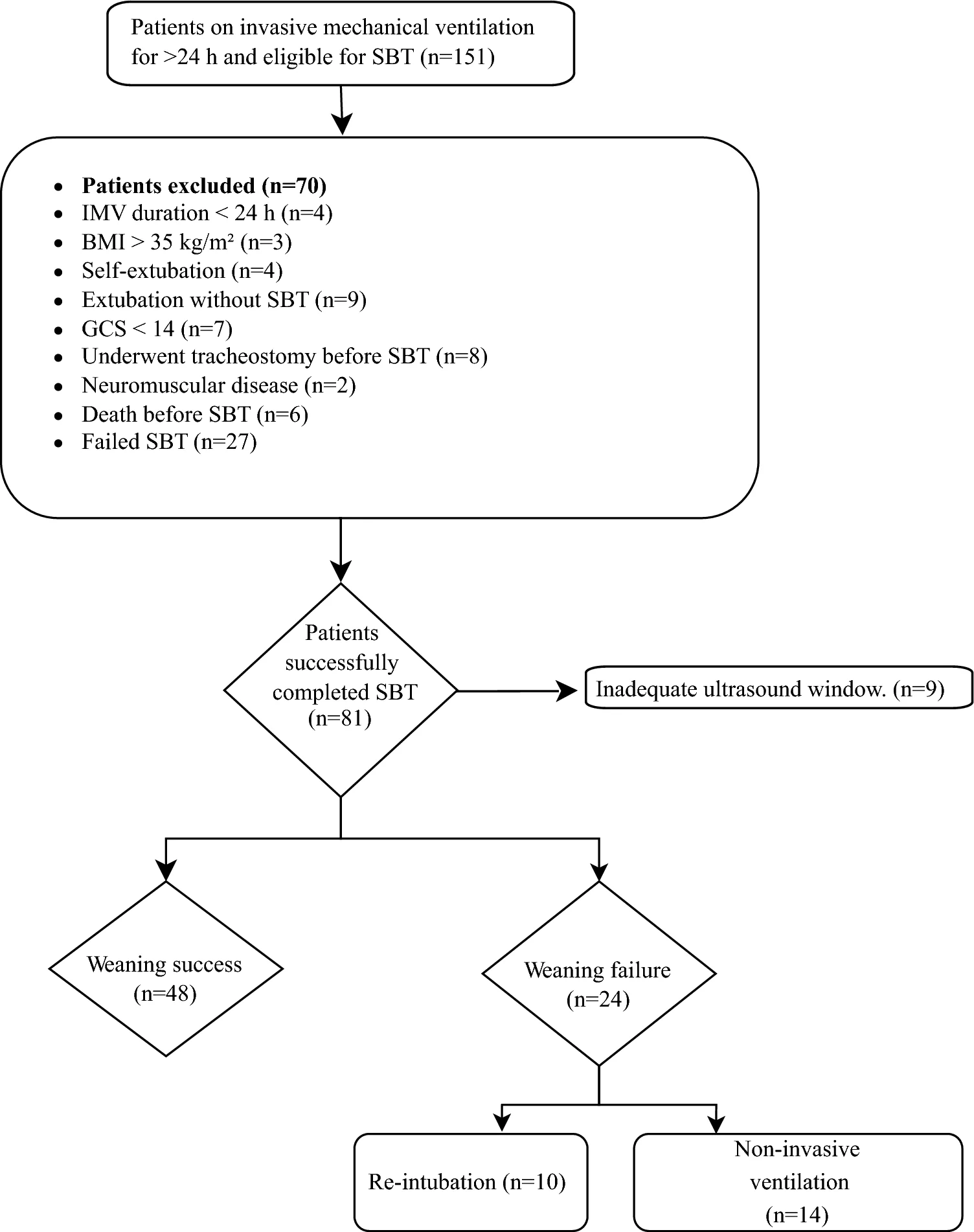
Flowchart of patients included in and excluded from the study. *IMV* invasive mechanical ventilation, *BMI* body mass ındex, *GKS* Glasgow Coma Scale, *PEEP* positive end-expiratory pressure, *NIV* non-invasive ventilation, *SBT* spontaneous breathing trial, *ICU* intensive care unit

A total of 151 ICU patients on mechanical ventilation were assessed, and 43 were excluded (4 had IMV duration < 24 h; 3 had BMI > 35 kg/m^2^; 4 self-extubated; 9 were extubated without an SBT; 7 had GCS < 14; 8 underwent tracheostomy; 2 had neuromuscular disease; 6 died before SBT). Among the remaining 108 patients, 81 successfully completed the SBT. In 9 patients, the diaphragm could not be visualized on ultrasound during the SBT. Thus, 72 patients met all inclusion criteria and were analyzed: 48 had successful extubations and 24 had extubation failures. The ICU admission diagnoses for these patients are listed in Table 1. Among the extubation failure group, 10 patients required reintubation and 14 required non-invasive ventilation (NIV) (Fig. 2).

**Table 1:** Admission diagnoses to the intensive care unit by group

Primary hospitalization diagnosis	Successful extubation	Failed extubation
	*n* (%)	*n* (%)
Acute pancreatitis	2 (%4.2)	0 (%0)
Acute renal failure	2 (%4.2)	2 (%8.3)
Diabetic ketoacidosis	0 (%0)	1 (%4.2)
Po. Fournier’s gangrene	1 (%2.1)	0 (%0)
Gastrointestinal bleeding	3 (%6.3)	0 (%0)
Epidural hematoma	1 (%2.1)	0 (%0)
Acute ischemic stroke	3 (%6.3)	2 (%8.3)
Acute exacerbation of COPD	2 (%4.2)	1 (%4.2)
Po. pancreatic cancer	1 (%2.1)	0 (%0)
Acute heart failure	0 (%0)	1 (%4.2)
Pneumonia	14 (%29.2)	9 (%37.5)
Po. gastric cancer	1 (%2.1)	0 (%0)
Po. ileus	6 (%12.5)	2 (%8.3)
Po. colon cancer	1 (%2.1)	0 (%0)
Po. femur fracture	4 (%8.3)	0 (%0)
Acute pulmonary embolism	1 (%2.1)	0 (%0)
Subarachnoid hemorrhage	2 (%4.2)	1 (%4.2)
Sepsis/septic shock	2 (%4.2)	4 (%16.7)
Acute liver failure	1 (%2.1)	0 (%0)
Status epilepticus	1 (%2.1)	1 (%4.2)

*PO* postoperative, *COPD* chronic obstructive pulmonary disease

There were no significant differences between the successful and failed extubation groups in age, sex, or SOFA score. The intubation duration was significantly longer in the failure group (mean ± SD: 10.75 ± 5.38 vs. 7.19 ± 4.93 days; *p* = 0.007). Respiratory rate (RR), tidal volume (TV), minute ventilation (MV), P0.1, and RSBI did not differ significantly between the 2 groups. Dynamic compliance (Cdyn) was significantly lower in the extubation failure group compared to the success group (34.92 ± 8.76 vs. 41.42 ± 12.13 mL/cmH_2_O, *p* = 0.025) (Tables 2, 3).

**Table 2:** Demographic features of the groups

	Successful extubation (*n* = 48)	Failed extubation (*n* = 24)	*p*
Age _mean ± SD_	64.65 ± 15.98	69.92 ± 10.54	^1^0.093
Gender _*n* (%)_			
Male	26 (%54.2)	10 (%41.7)	^2^0.453
Woman	22 (%45.8)	14 (%58.3)	

^1^Student *t* test

^2^Continuity (Yates) correction *p* < 0.05

**Table 3:** Comparison of body mass ındex values according to extubation success and visualization of diaphragm ultrasound ımaging

		BMI (kg/m^2^)		95% CI	
		Mean ± SD	Cohen’s d	Lower	Upper
Extubation	Successful	26.24 ± 4.63	− 0.39	− 0.883	0.106
	Failed	27.98 ± 4.10			
	*p*	0.123			
Visualization	Adequate	26.82 ± 4.50	− 0.783	− 1.484	− 0.077
	İnadequate	30.44 ± 5.61			
	*p*	0.030*			

Student *t* test*p < 0.05

*BMI* body mass ındex

**Fig. 3:**
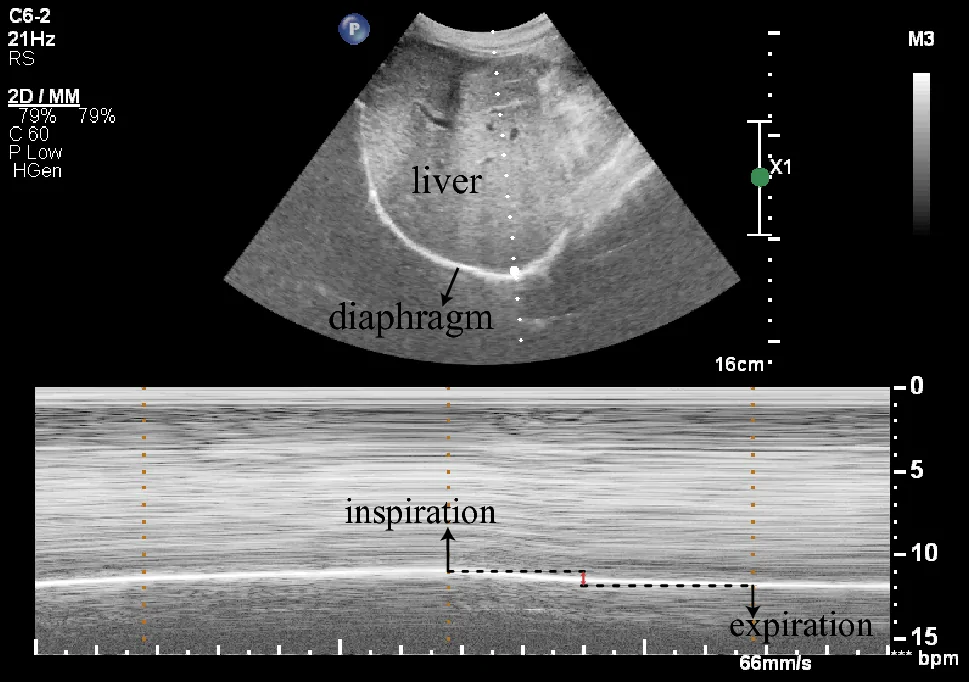
Ultrasonographic assessment of diaphragmatic excursion. The upper part shows B-mode image providing an anatomical view of the diaphragm. The lower part displays M-mode tracing, which depicts the amplitude and pattern of diaphragmatic motion over time during respiration

Laboratory values on the day of extubation were similar between groups. There were no significant differences in serum phosphorus, total calcium, ionized calcium, or magnesium levels between the success and failure groups (Table 4) (Fig. 3).

**Table 4:** Comparison of parameters used in intensive care unit and mechanical ventilation values

	Successful extubation		Failed extubation		
	Min–max	Mean ± SD (Median)	Min–max	Mean ± SD (Median)	*p*
Intubation duration (day)	1–19	7.19 ± 4.93 (7)	2–19	10.75 ± 5.38 (12)	0.007*
SOFA	1–5	2.27 ± 1.11 (2)	1–5	2.13 ± 1.12 (2)	0.576
Respiratory rate (breaths/min)	10–25	16.25 ± 3.62 (16)	9–23	15.25 ± 3.45 (15)	0.336
Tidal volume (L)	0.29–0.63	0.5 ± 0.07 (0.5)	0.38–0.65	0.49 ± 0.06 (0.5)	^+^0.664
RSBI (breaths/min/L)	19.23–72.41	33.44 ± 10.09 (31.5)	18–52.38	31.7 ± 8.67 (32)	0.591
Minute volume (L/min)	4.4–15.5	8.08 ± 2.24 (7.6)	4.5–10.81	7.45 ± 1.83 (7.4)	0.345
P0.1 (cmH_2_O)	0.1–4.3	1 ± 0.86 (0.6)	0.1–2.1	0.92 ± 0.51 (0.9)	0.649
Cdyn (ml/cmH_2_O)	24–78	41.42 ± 12.13 (40)	22–57	34.92 ± 8.76 (33.5)	0.025*

Mann–Whitney *U* test

*RSBI* rapid shallow breathing index, *P0*.*1* airway occlusion pressure, *Cdyn* dynamic compliance, *L* liter, *Min* minute

*p < 0.05

^+^Student’s *t* test

Regarding diaphragm ultrasound parameters, DE and DTF were significantly higher in the successful extubation group {DE: 1.946 ± 0.428 cm vs. 1.502 ± 0.464 cm, AUC 0.750 (*p* = 0.001)}; {DTF: 33.58 ± 9.91% vs. 25.37 ± 8.1%, AUC 0.756 (*p* = 0.001)}, whereas D-RSBI was significantly lower {8.89 ± 3.46 vs. 11.51 ± 5.08, AUC 0.649 (*p* = 0.040)}. There was no significant difference between the 2 groups in terms of the RSDI. The semi-quantitative cough strength score (SCSS) was significantly higher in the successful extubation group (Tables 5, 6).

**Table 5:** Comparison of laboratory parameters

	Successful extubation		Failed extubation		
	Min–max	Mean ± SD	Min–max	Mean ± SD	*p*
Phosphorus (mg/dl)	0.8–6.48	3.12 ± 1.32	1.41–4.52	2.85 ± 0.84	0.371
Total calcium (mg/dl)	7–10.3	8.02 ± 0.77	6.8–9.79	8.11 ± 0.79	0.632
Ionized calcium (mmol/L)	0.76–1.38	1.03 ± 0.12	0.66–1.29	1.05 ± 0.15	0.545
Magnesium (mg/dl)	0.7–2.51	1.77 ± 0.41	1.35–2.86	1.88 ± 0.39	0.279

Student’s *t* test *p < 0.05

*SD* standard deviation

**Table 6:** Evaluation of groups in terms of diaphragm measurements

	Successful extubation		Failed extubation		
	Min–max	Mean ± SD (median)	Min–max	Mean ± SD (median)	*p*
DE (cm)	1.1–2.6	1.946 ± 0.428 (2.05)	0.92–2.4	1.502 ± 0.464 (1.3)	0.001*
DTF (%)	16.67–61.54	33.58 ± 9.91 (34.2)	18.18–50	25.37 ± 8.1 (22.2)	0.001*
D-RSBI	4.8–21.82	8.89 ± 3.46 (8.5)	4.09–22.45	11.51 ± 5.08 (12.7)	0.040*
RSDI	9.19–38.28	18.11 ± 6.85 (16.6)	7.91–53.45	24.06 ± 11.79 (22.4)	0.054

Mann Whitney *U* test *p < 0.05

*DE* diaphragm excursion, *DTF* diaphragm thickness fraction, *D*-*RSBI* diaphragmatic rapid shallow breathing index, *RSDI* rapid shallow diaphragmatic index, *SD* standard deviation, *cm* centimeter

DTF showed a moderate, statistically significant negative correlation with tidal volume (TV) (*r* = –0.500, *p* = 0.001) (Table 7).

**Table 7:** ROC analysis results for diaphragm measurements and SCSS in the diagnosis of failed extubation

	AUC	SE	95% CI	*p*	Cut-off point	Sensitivity	Specificity
DE (cm)	0.750	0.06	0.634–0.845	0.001*	≤ 1.85	83.3	66.7
DTF (%)	0.756	0.06	0.640–0.849	0.001*	≤ 23.5	66.7	81.3
D-RSBI	0.649	0.08	0.528–0.758	0.040*	> 11.05	58.3	87.5
RSDI	0.640	0.08	0.518–0.750	0.075	–		
SCSS	0.704	0.06	0.584–0.805	0.001*	≤ 3	83.3	43.7

*AUC* area under curve, *SE* standart error, *CI* confidence interval, *DE* diaphragm excursion, *DTF* diaphragm thickness fraction, *D*-*RSBI* diaphragmatic rapid shallow breathing index, *RSDI* rapid shallow diaphragmatic index, *SCSS* semi-quantitative cough strength score, *cm* centimeter

Multivariate logistic regression model was found to be statistically significant (*p* < 0.001), the Nagelkerke R^2^ was 0.488, and the explanatory coefficient of the model (%83.3) was at a good level. The analysis identified three independent predictors of successful extubation: dynamic compliance (Cdyn; OR: 1.094), diaphragmatic thickening fraction (DTF; OR: 1.109), and diaphragm excursion (DE; OR: 10.018) (Table 8).

**Table 8:** Correlations between diaphragm ultrasound measurements and IMV parameters

	DE (cm)	TDI (cm)	TDE (cm)	DTF (%)	D-RSBI	RSDI
Respiratory rate (breaths/min)	*r* − 0.219	0.231	0.209	− 0.119	0.670	0.652
	*p* 0.064	0.051	0.078	0.319	0.001*	0.001*
^+^Tidal volume (L)	*r* − 0.073	0.213	0.428	− 0.500	0.071	− 0.297
	*p* 0.545	0.073	0.001*	0.001*	0.554	0.011*
Minute volume (L/min)	*r* − 0.176	0.296	0.376	− 0.296	0.572	0.406
	*p* 0.139	0.012*	0.001*	0.011*	0.001*	0.001*
P0.1 (cmH_2_O)	*r* − 0.183	0.253	0.337	− 0.268	0.295	0.183
	*p* 0.124	0.032*	0.004*	0.023*	0.012*	0.123
Cdyn (ml/cmH_2_O)	*r* − 0.004	0.155	0.115	0.010	0.020	− 0.161
	*p* 0.975	0.195	0.337	0.932	0.865	0.177

Spearman’s rho correlation analysis

*P0*.*1* airway occlusion pressure, *Cdyn* dynamic compliance, *TDI* diaphragm thickness at end-inspiration, *TDE* diaphragm thickness at end-expiration, *DE* diaphragm excursion, *DTF* diaphragm thickness fraction, *D*-*RSBI* diaphragmatic rapid shallow breathing index, *RSDI* rapid shallow diaphragmatic index, *cm* centimeter

^+^Pearson correlation analysis

*p < 0.05

**Fig. 4:**
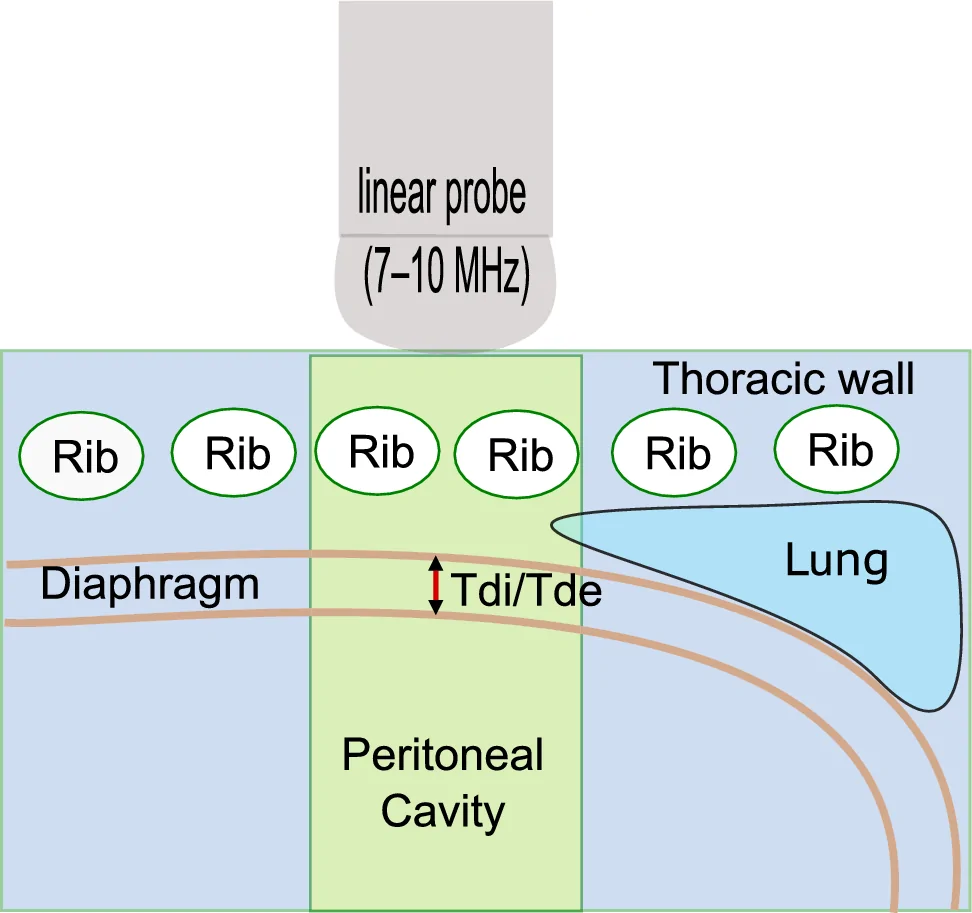
Illustration of the technique for measuring diaphragm thickness and display of anatomical structures in the zone of apposition

The intra-observer reliability for ultrasound parameters was: DE (ICC = 0.932, 95% CI 0.870–0.965), DTF (ICC = 0.961, 95% CI 0.923–0.980), TDI (ICC = 0.894, 95% CI 0.800–0.946), and TDE (ICC = 0.868, 95% CI 0.755–0.932) [Supplementary Table 1] (Fig. 4).

## Discussion

**Fig. 5:**
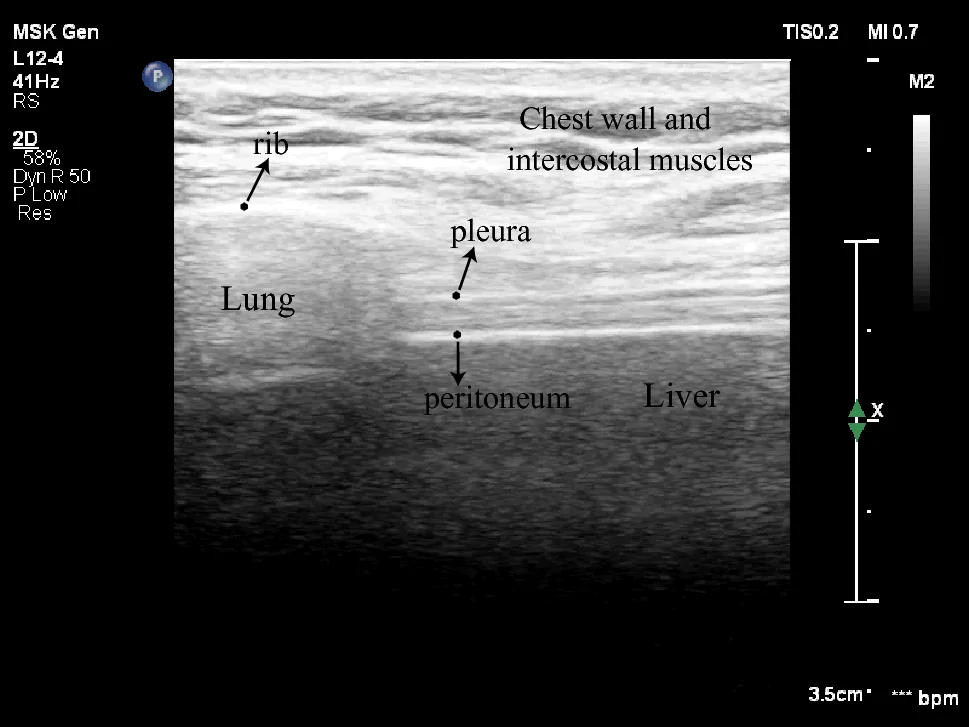
B-mode ultrasonographic image of the diaphragm at the zone of apposition. The image displays the characteristic trilaminar structure of the diaphragm for measuring diaphragm thickness, consisting of a central hypoechoic muscle layer bordered by 2 hyperechoic lines corresponding to the diaphragmatic pleura and peritoneum (indicated by the arrow)

Diaphragmatic excursion (DE) and diaphragm thickening fraction (DTF) are the main ultrasonographic parameters used to assess the diaphragm’s displacement and contractility functions, respectively. Our study shows that both diaphragm excursion (DE, AUC (Area under the curve): 0.750, *p* = 0.001) and diaphragm thickening fraction (DTF, AUC: 0.756, *p* = 0.001) have a statistically significant and independent role in predicting extubation success. When we compared the results with the recent systematic review and meta-analysis, the sensitivity (83.3%) and specificity (66.7%) values we found for DE were remarkably similar to the pooled values (79.9 and 69%) reported by Mahmoodpoor et al. For DTF, the specificity of 81.3% in our study is consistent with the findings of both meta-analyses (75 and 81%). However, the sensitivity of 66.7% in our study is lower than the values reported in the meta-analyses (85 and 89%). We believe that this difference is due to the heterogeneous studies including both PSV and T-tube trials in both meta-analyses, whereas in our study, patients were evaluated under fixed PEEP and pressure support levels during spontaneous breathing trials (SBTs), which is one of the major methodological limitations in prior studies. Since PSV inherently reduces the patient’s respiratory workload, this can reduce the sensitivity of DTF as a test, leading to falsely reassuring (false-negative) DTF values despite underlying diaphragmatic dysfunction. In other words, PSV can mask diaphragmatic weakness by offloading work of breathing, thereby reducing DTF’s sensitivity for detecting extubation failure. Furthermore, although subgroup analysis by Mahmoodpoor et al. showed that the diagnostic accuracy of DTF was higher in T-tube trials than in PSV trials. In contrast, Parada-Gereda et al. [11, 16] found no significant difference in the sensitivity or specificity of DE and DTF between both trials (Fig. 5).

Respiratory rate and RSBI are commonly used indicators of weaning readiness, but they are non-specific and can be influenced by compensatory mechanisms and neither parameter was found to be statistically significant in our study. Incorporating diaphragmatic function into the assessment of these indices can provide valuable insight into the cause of respiratory distress. For instance, patients with diaphragmatic dysfunction may appear to have an acceptable RSBI using accessory respiratory muscles; however, because these auxiliary muscles fatigue faster than the diaphragm, such patients often develop respiratory failure soon after extubation despite a “normal” RSBI. To overcome this diagnostic gap, new indexes D-RSBI = RR/DE and RSDI = RSBI/DE were proposed by including diaphragmatic measurements into the RSBI [17, 18].

D-RSBI replaces tidal volume with DE in the RSBI formula, since tidal volume can be kept within normal ranges by compensatory mechanisms. Spadaro et al. (using T-tube trials, AUC = 0.89) and Song et al. [17, 19] (using PSV trials, AUC = 0.813) reported that D-RSBI had higher diagnostic accuracy than conventional RSBI. We believe that the relatively lower AUC in our study (0.652) is due to our exclusion of patients with severe diaphragmatic dysfunction who could not complete the SBT.

RSDI is an index that evaluates the association between RSBI and diaphragmatic function. In our study, neither RSDI nor RSBI proved to be a significant predictor (*p* = 0.075 and *p* = 0.591, respectively), consistent with the findings of Fossat et al. [20] in patients who passed an SBT under PSV.

In our analysis, D-RSBI and RSDI (both of which include respiratory rate in their formula) were unsurprisingly found to correlate strongly with RR and minute ventilation. However, even without a direct mathematical linkage, DTF showed a moderate, statistically significant negative correlation with tidal volume, suggesting a possible physiological relationship. The negative correlation we observed is consistent with the pathophysiology of ventilator-induced diaphragm dysfunction (VIDD). High levels of ventilatory support produce larger tidal volumes in normal lungs, but also inhibit the patient’s respiratory effort and thus the strength of diaphragmatic contraction. This clinically recognized process, known as over-assistance myotrauma (disuse atrophy), plays a central role in the development of VIDD and is characterized by a reduced DTF value [4].

We found a statistically significant correlation (*r* = − 0.268, *p* = 0.023) between DTF and P0.1, which we examined as a correlation pair. In a study by Umbrello et al. [21] excluding patients with chronic obstructive pulmonary disease (COPD) and high PEEP pressure, DTF and P0.1 correlated (*b* = 5.522, *p* < 0.001). However, Schulz et al. [22] reported that in patients with COPD, the central drive due to hyperinflation and flattening of the diaphragm would not result in the same rate of diaphragm contraction. Furthermore, Formenti et al. reported that the correlation between DTF and respiratory effort assessed by esophageal pressure swing was very strong at PEEP 0 cmH_2_O (*R*^2^ = 0.871) but became statistically insignificant at PEEP 15 cmH_2_O (*p* = 0.066) [23]. In total, the strength of this correlation is influenced by different clinical and methodological factors, including the heterogeneity of the patient population, underlying disease (especially COPD), ventilator settings (specifically PEEP level). Since P0.1 evaluates central respiratory drive while DTF reflects the peripheral muscular response, these 2 parameters should be used together rather than interchangeably. Clinical context (e.g., underlying COPD or ventilator settings) should be considered when interpreting their values.

Dynamic compliance provides valuable information about the elasticity of the respiratory system and airway resistance. Dynamic compliance was significantly lower in the extubation failure group and was found to be an independent risk factor in our logistic regression analysis (OR: 1.094, 95% CI 1.022–1.171, *p* = 0.010) [24]. More importantly, there was no statistically significant correlation between diaphragm ultrasound (DUS) and dynamic lung compliance. This finding supports the recommendation that lung ventilation and diaphragm function be evaluated together as complementary aspects of respiratory mechanics, consistent with reports in the literature [25].

BMI was not associated with the outcome of extubation (*p* = 0.123) (Table 9). Consistent with the literature, the mean BMI of patients with inadequate imaging was significantly higher than that of patients with adequate imaging (30.44 ± 5.61 vs. 26.82 ± 4.50 kg/m^2^; *p* = 0.030) [26]. Due to the small number of patients in this subgroup (*n* = 9) that an image could not be obtained with diaphragm ultrasound, prospective studies including different BMI classifications are needed to confirm this relationship and its impact on clinical practice.

**Table 9:** Evaluation of the parameters after excluding D-RSBI to predict extubation success via logistic regression analysis

	OR	95% CI for EXP(B)		*p*
		Lower	Upper	
DTF (%)	1.109	1.027	1.198	0.009*
Cdyn (ml/cmH_2_O)	1.094	1.022	1.171	0.010*
DE (cm)	10.018	2.198	45.662	0.003*

*OR* odds ratio, CI confidence interval, *Cdyn* dynamic compliance, *DE* diaphragm excursion, *DTF* diaphragm thickness fraction, *D*-*RSBI* diaphragmatic rapid shallow breathing index, *cm* centimeter

Serum electrolytes are critical for neuromuscular transmission and muscle contraction. However, there is insufficient data in the literature regarding the role of electrolytes in the weaning process from mechanical ventilation. A previous study found that hypophosphatemia at the time of admission to the intensive care unit (ICU) was associated with prolonged mechanical ventilation [27]. Consistent with the findings of our current study, another study revealed that phosphorus, calcium, and magnesium levels did not play a significant role in predicting the success of weaning from the ventilator [28].

Inadequate cough strength and inability to clear secretions are well-known risk factors for extubation failure. In our study, SCSS was statistically significant in determining weaning success in accordance with the literature [29]. Although SCSS was not retained as an independent predictor in the final model, its moderate AUC (0.704) and high sensitivity suggest potential value as a screening tool, especially in patients at risk for secretion retention.

Prior to creating the multivariate model, we assessed for multicollinearity among diaphragmatic ultrasound parameters (DE, DTF, and D-RSBI) that were statistically significant in predicting extubation success. Variance ınflation factor (VIF) and condition ındex analyses revealed no significant multicollinearity [Supplementary Table 2]. However, a strong correlation was observed between D-RSBI and DE [Supplementary Table 3]. This finding is expected as D-RSBI is mathematically derived from DE. The DeLong test confirmed that, although both parameters were statistically significant, DE showed statistically significant higher AUC (area under curve) [Supplementary Table 4, Supplementary Fig. 1]. In contrast to the previous study conducted with pediatric patients [2], we included DE in our regression model due to its higher AUC. Consequently, D-RSBI was excluded from the logistic regression model to improve model stability and parsimony [Supplementary Table 5].

The final backward-stepwise logistic regression model (including DE, DTF, SCSS, Cdyn, and intubation duration) was statistically significant (*p* < 0.001). This model yielded a Nagelkerke R^2^ of 0.488 and an overall correct classification rate of 83.3%, indicating good predictive power. This analysis identified three independent predictors of successful extubation: dynamic compliance (Cdyn, OR 1.094), diaphragmatic thickening fraction (DTF, OR 1.109), and most notably, diaphragm excursion (DE, OR 10.018). Variance Inflation Factor (VIF) values and condition ındices confirmed absence of significant multicollinearity among these variables. Consequently, the substantial odds ratio for DE highlights its dominant role as a robust and independent predictor of weaning outcome (Tables 8–10).

**Table 10:** Multicollinearity assessment of independent predictors of successful extubation for the logistic regression model

	VIF	Condition index	Eigenvalue
DE (cm)	1.07	2.91	1.32
DTF (%)	1.08	2.85	1.31
Cdyn	1.01	2.99	1.00

*VIF* Variance Inflation Factor, *DE* diaphragm excursion, *DTF* diaphragm thickness fraction, *Cdyn* dynamic compliance, *cm* centimeter

### Strengths and limitations

Our results support that extubation success is multifactorial and best predicted by integrating muscle function, respiratory mechanics, and airway protection. The strengths of this study include its prospective design, a standardized ultrasound methodology: all patients were assessed with the same technique (fixed ventilator support settings, consistent patient positioning, same side/approach for DUS). We also included only neurologically intact patients (GCS > 14) with intact airway protection reflexes. This design enabled us to isolate the role of diaphragm function. Ultrasound measurements performed by a single, experienced operator have minimized inter-observer variability. Limitations include a single-center design and modest sample size. Focusing on neurologically stable patients limits the generalizability of the study. There is no standardization in terms of mechanical ventilation modes, PEEP levels, and neuromuscular blocker use in patients undergoing controlled mechanical ventilation prior to SBT, which may affect diaphragm function. The interval between the SBT and extubation was not fixed, which may have contributed to diaphragmatic muscle fatigue. Moreover, we did not perform a detailed cardiac assessment prior to extubation to rule out cardiac dysfunction as a contributor to weaning failure.

Despite consistent statistical significance in Bootstrap and penalized regression analyses as well as in univariate logistic regression, the relatively wide confidence interval of the DE suggests that the effect size may be affected by limited sample size and physiological interrelationships of diaphragm measurements. [Supplementary Tables 6–8]. Therefore, the effect size of DE should be interpreted with appropriate caution until it is validated in larger standardized multi-center cohorts [30]. Finally, ultrasound measurements are operator-dependent. Since our study was conducted by a single operator, inter-observer reproducibility for ultrasound measurements was not assessed.

### Future directions

Future studies should use standardized protocols (e.g., fixed PEEP and pressure support settings) and focus on specific patient subgroups (such as neurological or ARDS patients), ideally with multi-center enrollment and external validation, to further delineate the role of diaphragm ultrasound in extubation success. Studies enrolling all intensive care patients who are planned for extubation are needed to improve the generalizability of findings. In addition, incorporating diaphragm ultrasound into multivariate predictive models alongside other non-invasive assessments (IMV parameters, accessory respiratory muscles, extra-diaphragmatic muscles) may further enhance predictive accuracy. By combining traditional indices with diaphragmatic, cardiac, and neurological assessments, a comprehensive tool or scoring system can be created to guide extubation decisions. Furthermore, by understanding how respiratory system (respiratory muscles, lungs, central drive, etc.) interact, we can improve our understanding of the respiratory pathophysiology for successful extubation.

## Conclusion

In this prospective observational study, diaphragm ultrasound demonstrated higher predictive value than traditional weaning indices for extubation outcomes in neurologically intact intensive care patients receiving invasive mechanical ventilation. Among the evaluated ultrasonographic parameters, diaphragm excursion and diaphragm thickening fraction emerged as robust and independent predictors of extubation success, whereas traditional indices such as the rapid shallow breathing index alone could not distinguish between successful and unsuccessful extubations. Importantly, the integration of diaphragm ultrasound parameters with respiratory system mechanics, particularly dynamic compliance, improved overall predictive performance, highlighting the multifactorial nature of extubation readiness. These findings suggest that diaphragm ultrasound provides clinically relevant, complementary information beyond standard weaning criteria and may improve the extubation decision-making process when implemented within a standard assessment protocol.

## Supplementary Information

Below is the link to the electronic supplementary material.

Supplementary file1 (DOCX 81 KB)

## Data Availability

The data that support the findings of this study are available from the corresponding author upon reasonable request.
