# Supplementary material for: Comparison of standardized diaphragm ultrasound measurement and conventional methods for predicting weaning failure: a prospective observational study
Source: J Anesth. 2026 Jan 24;40(5):695–707. doi: 10.1007/s00540-026-03655-6 (PMC13624037; doi:10.1007/s00540-026-03655-6)
Supplement: Supplementary file 1 [file 540_2026_3655_MOESM1_ESM.docx]

**Table 1: Intra-observer Reliability of the Ultrasound Measurements**

|  | **ICC** | **95% CI** | | **PooledSD** | **SEM** | **MDC** |
| --- | --- | --- | --- | --- | --- | --- |
| TDI (cm) | 0.894 | 0.800 | 0.946 | 0.034 | 0.011 | 0.030 |
| DTE (cm) | 0.868 | 0.755 | 0.932 | 0.022 | 0.008 | 0.022 |
| DTF (%) | 0.961 | 0.923 | 0.980 | 16.398 | 3.251 | 9.012 |
| DE (cm) | 0.932 | 0.870 | 0.965 | 0.320 | 0.083 | 0.231 |

ICC Intraclass Correlation Coefficient, 95% CI 95% Confidence Interval, PooledSD Pooled Standard Deviation, SEM Standard Error of Measurement, MDC Minimal Detectable Change, TDI Diaphragm thickness at end-inspiration,TDE Diaphragm thickness at end-expiration, DE diaphragm excursion, DTF diaphragm thickness fraction, cm centimeter.

**Table 2: Multicollinearity Assessment of Diaphragm Ultrasound Parameters Statistically Significant in Extubation Success**

|  | **VIF** | **Condition Index** | **Eigenvalue** |
| --- | --- | --- | --- |
| **DE (cm)** | 2.95 | 1.00 | 1.96 |
| **DTF (%)** | 1.09 | 1.52 | 0.85 |
| **D-RSBI** | 3.00 | 3.24 | 0.19 |

*VIF: Variance Inflation Factor*

DE diaphragm excursion, DTF diaphragm thickness fraction, D-RSBI diaphragmatic rapid shallow breathing index, cm centimeter.

**Table 3: Correlation analysis of diaphragm ultrasound parameters statistically significant in extubation success**

|  | **DE (cm)** | **DTF (%)** | **D-RSBI** |
| --- | --- | --- | --- |
| **DE (cm)** | 1.00 | 0.229 (p = 0.053) | –0.834 (p < 0.001) |
| **DTF (%)** | 0.229 (p = 0.053) | 1.00 | –0.181 (p = 0.128) |
| **D-RSBI** | –0.834 (p < 0.001) | –0.181 (p = 0.128) | 1.00 |

*Spearman’srhokorelasyon analizi	^+^Pearson korelasyon analizi	*p<0.05*

DE diaphragm excursion, DTF diaphragm thickness fraction, D-RSBI diaphragmatic rapid shallow breathing index, cm centimeter.

**Table 4: ROC analysis results for diaphragm measurements in the diagnosis of failed extubation**

|  | **AUC** | **SE** | **95% CI** | **Delong test p** |
| --- | --- | --- | --- | --- |
| DE (cm) | 0.750 | 0.06 | 0.634-0.845 | 0.008* |
| D-RSBI | 0.649 | 0.08 | 0.528-0.758 |  |

*AUC: Area Under Curve	SE: Standart error	CI: Confidence interval	*p<0.05*

DE diaphragm excursion, D-RSBI diaphragmatic rapid shallow breathing index, cm centimeter.

**Figure 1: ROC curves of DE and D-RSBI parameters**

**Table 5: Multicollinearity assessment of the remaining parameters for the logistic regression model after excluding D-RSBI**

|  | **VIF** | **Condition Index** | **Eigenvalue** |
| --- | --- | --- | --- |
| **DE (cm)** | 1.36 | 1.00 | 2.11 |
| **DTF (%)** | 1.25 | 1.45 | 1.00 |
| **Cdyn** | 1.01 | 1.74 | 0.69 |
| **İntubation duration** | 1.23 | 1.67 | 0.76 |
| **SCSS** | 1.66 | 2.20 | 0.44 |

*VIF: Variance Inflation Factor*

DE diaphragm excursion, DTF diaphragm thickness fraction, D-RSBI diaphragmatic rapid shallow breathing index, Cdyn dynamic compliance, SCSS semiquantitative cough strength score, cm centimeter.

**Table 6: Predictors of successful extubation identified by penalized logistic regression models (Ridge and LASSO)**

|  | **Original OR** | **Ridge OR** | **LASSO OR** |
| --- | --- | --- | --- |
| **DE (cm)** | 10.018 | 6.802 | 6.781 |
| **DTF (%)** | 1.109 | 1.087 | 1.083 |
| **Cdyn** | 1.094 | 1.071 | 1.064 |

*OR: Odds ratio*

DE diaphragm excursion, DTF diaphragm thickness fraction, Cdyn dynamic compliance, cm centimeter.

**Table 7 : Bootstrap internal validation of the logistic regression model for extubation success (1000 Resamples)**

|  | **Original Model** | **Bootstrap-Corrected** | |
| --- | --- | --- | --- |
| **AUC** | 0.864 | 0.842 |  |
| **Brier Score** | 0.120 | 0.128 |  |

*AUC: Area Under Curve*

**Table 8: Factors associated with successful extubation: Univariate logistic regression analysis.**

|  | **OR** | **95% CI** | **p** |
| --- | --- | --- | --- |
| **DE (cm)** | 8.204 | 2.482 – 27.144 | 0.001* |
| **DTF (%)** | 1.118 | 1.042 – 1.199 | 0.001* |
| **Cdyn** | 1.064 | 1.011 – 1.119 | 0.027* |

*OR: Odds ratio	CI: Confidence interval	*p<0.05*

DE diaphragm excursion, DTF diaphragm thickness fraction, Cdyn dynamic compliance, cm centimeter.
